# Antiferromagnetic spintronics: An overview and outlook

**DOI:** 10.1016/j.fmre.2022.03.016

**Published:** 2022-04-08

**Authors:** Danrong Xiong, Yuhao Jiang, Kewen Shi, Ao Du, Yuxuan Yao, Zongxia Guo, Daoqian Zhu, Kaihua Cao, Shouzhong Peng, Wenlong Cai, Dapeng Zhu, Weisheng Zhao

**Affiliations:** a Fert Beijing Institute, MIIT Key Laboratory of Spintronics, School of Integrated Circuit Science and Engineering, Beihang University, Beijing 100191, China; b Beihang-Goertek Joint Microelectronics Institute, Qingdao Research Institute, Beihang University, Qingdao 266000, China

**Keywords:** Antiferromagnets, MRAM, Spintronics, Spin-orbit torque, Exchange bias

## Abstract

Over the past few decades, the diversified development of antiferromagnetic spintronics has made antiferromagnets (AFMs) interesting and very useful. After tough challenges, the applications of AFMs in electronic devices have transitioned from focusing on the interface coupling features to achieving the manipulation and detection of AFMs. As AFMs are internally magnetic, taking full use of AFMs for information storage has been the main target of research. In this paper, we provide a comprehensive description of AFM spintronics applications from the interface coupling, read-out operations, and writing manipulations perspective. We examine the early use of AFMs in magnetic recordings and conventional magnetoresistive random-access memory (MRAM), and review the latest mechanisms of the manipulation and detection of AFMs. Finally, based on exchange bias (EB) manipulation, a high-performance EB-MRAM is introduced as the next generation of AFM-based memories, which provides an effective method for read-out and writing of AFMs and opens a new era for AFM spintronics.

## 1 Introduction

Recently, antiferromagnets (AFMs) have drawn great interest in the field of spintronics and are considered the next generation of magnetic memories owing to their novel advantages over conventional ferromagnets (FMs), such as the absence of stray fields, high intrinsic precession frequency (∼ THz), and high stability under magnetic fields [1]. Different from FMs, AFMs generally refer to two coupled sublattices with spontaneous magnetic moments in opposite directions. These spontaneous magnetic moments **m_A_** and **m_B_** counterbalance and do not exhibit macroscopic magnetism. Since the Nobel lecture of L. Néel in the 1970s, AFMs were considered “interesting but useless” [2]. After more than 20 years of development, AFMs were first applied to giant magnetoresistance (GMR) heads [3], directly driving innovation in the hard disk industry since 1997, which then made AFMs interesting and useful. For instance, within the next 20 years, with the advent of magnetoresistive random-access memory (MRAM), from the early Toggle-MRAM, spin-transfer torque (STT) -MRAM, thermally-assisted (TA) -MRAM, to spin-orbit torque (SOT) -MRAM, AFMs and synthetic antiferromagnets (SAFs) have become popular in spintronics devices [4], [5], [6], [7].

However, in the past, the applications of AFMs have mainly focused on the strong interface coupling between AFMs and FMs. The information carrier is still an FM, which does not take full advantage of the AFMs. Therefore, many efforts have focused on finding effective ways to better detect and manipulate the magnetic moment directions of AFMs, that is, the new physics or related effects of AFM spintronics [1,8]. There were no obvious responses to either electric or magnetic fields for the AFMs. Although AFMs exhibit anisotropic magnetoresistance (AMR) [9], spin Hall magnetoresistance (SMR) [10], anomalous Hall effect (AHE) [11,12], laser-induced anomalous Nernst effect [13], and magneto-optical Kerr effect (MOKE) [14,15], however, they are weak and exhibit low sensitivity in the readings. These limitations restrict the use of AFMs as carriers for information storage. Moreover, electrical manipulation of AFMs is challenging. Recent reports show that SOT can achieve switching of the Néel vectors in AFMs [9,16,17]. By applying a magnetic field or changing the temperature, researchers have clarified the magnetic origin of the resistance variation, which further confirms the switching of the AFMs [18,19]. Moreover, many efforts have been made to determine the mechanism of SOT-induced Néel vector switching, in which both the anti-damping torque and the thermoelastic effect contribute [20]. Although the writing operation of AFMs appears to have been established, the read-out of AFMs is still a limitation. The recently proposed EB-MRAM [21], combining SOT-induced EB switching [22] and high tunneling magnetoresistance (TMR), blazes a new path to the effective read-out and writing of the AFM and opens a new era for the next generation of AFM-based memories.

**Fig. 1:**
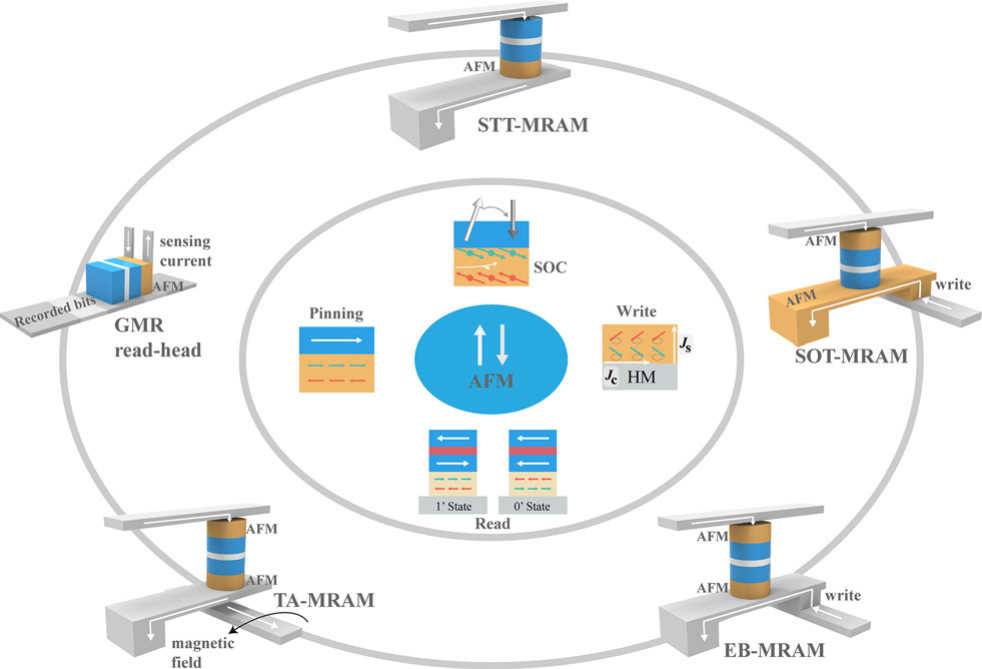
**Physical characterization and relevant applications in magnetic recording and memory based on antiferromagnet (AFM).** A schematic illustration of the relation between the intrinsic magnetic structure of AFM (inner ellipse) and the physical characterization when AFM is adjacent to ferromagnet (FM) or heavy metal (HM) (middle ellipse), such as pinning effect, spin-orbit torque (SOT), and the read and write of AFM. These properties can be used in magnetic recording and memories (outer ellipse), such as giant magnetoresistance (GMR) read-head, spin-transfer torque- (STT-), thermally-assisted- (TA-), spin-orbit torque- (SOT-), and exchange-bias- (EB-) magnetic random-access memory (MRAM).

In this paper, we present an overview of the typical progress in AFM-based applications in magnetic memories. Firstly, the main application of AFMs in the GMR head and MRAMs are discussed due to the strong interface coupling between AFMs and FMs, which generates an EB field (Fig. 1, left); followed by the spin-polarized properties of the novel AFMs as well as the EB fields, which may benefit field-free switching in SOT-MRAMs (Fig. 1, right). Moreover, we focus on the new effects of AFM spintronics, mainly on the current-induced manipulation of AFMs and their read-out techniques. Combining the read-out process from the magnetic tunnel junction (MTJ) and the writing operation from the current-induced EB switching, a possible new generation of AFM memory, EB-MRAM, is presented in detail (Fig. 1, bottom right). Finally, we propose a perspective on AFM spintronics and memory.

## 2 Application of AFM interface coupling

### 2.1 AFMs in magnetic recording

AFMs were described by Néel in his 1970 Nobel Prize speech as interesting but useless. After 20 years, AFMs were first used in a spin valve structure, which was at the heart of read heads in high-density hard drives. The AFMs acted as the reference layer. The introduction of AFMs enabled the large-scale application of the GMR effect, which expanded the horizon of magnetic recording [23,24]. The spin-valve structure soon brought about rapid advancement in the field of magnetic recording.

For most data-recording designs in the information field, magnetization has become a preferred option. The information can be stored by magnetization within a thin magnetic film or magnetic tape. A magnetic sensor can be used as a read head to read the data by detecting the fringing field a few nanometers above the storage medium. The early read head was based on the AMR. The resistance of FM metal varies when current flows at a different angle with its magnetization; thus, the data can be read via the resistance change. However, AMR was not significant at room temperature. It is usually a few percent and is not sensitive enough when the magnetic field is weak [3], especially when the storage density increases and the magnetic recording unit scales down.

**Fig. 2:**
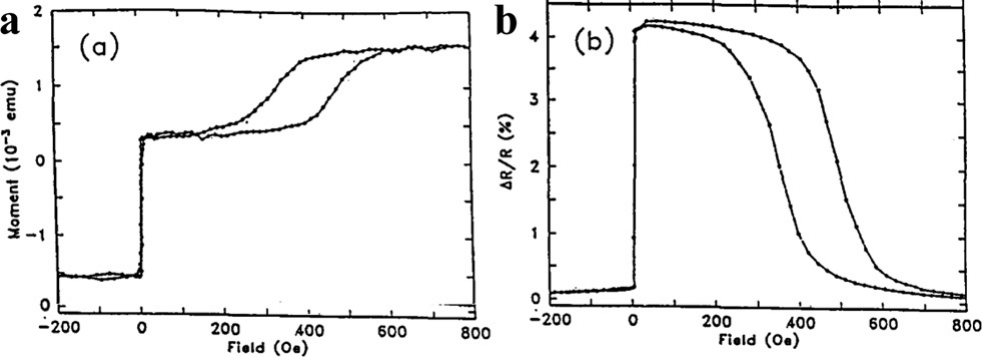
**Magnetic and electrical properties of a typical spin valve structure.** (a) Room-temperature hysteresis loop and (b) magnetoresistance of a sample Ta/NiFe/Cu/NiFe/FeMn/Ta [28].

The discovery of GMR represents a revolutionary breakthrough in storage technology. In 1988, Baibich et al. [25] and Binasch et al. [26] discovered the GMR effect in FM/nonmagnetic metal (NM)/FM multilayers, which won the Nobel Prize in 2007. The multilayers display low and high resistances when the two FM layers are parallel and antiparallel, respectively. The magnitude of the GMR ratio is much larger than that of the AMR, and the GMR is thus soon applied to the read head of a hard disk drive. In 1991, B. Dieny et al. proposed a device to achieve the practical use of GMR [3,27,28]. A sandwich structure consisting of two uncoupled FM layers separated by a nonmagnetic metal layer, which was termed as a spin valve, showed a magnetoresistance ratio of 5% in the range of only 10 Oe at room temperature as shown in Fig. 2. The first spin valve was achieved in the NiFe/Cu/NiFe/FeMn multilayers. The AFM FeMn layer was introduced to fix the magnetic moment of one FM layer; therefore, the FM layer was pinned in a certain direction by EB at the FM/AFM interface. Another FM layer can easily interact with the fringing magnetic field; hence, the parallel and antiparallel states of the two FM layers can be realized. AFMs provide a feasible solution for magnetic detection by GMR, which have led to the development of a hard disk drive.

### 2.2 AFMs in Toggle- and STT-MRAMs

**Fig. 3:**
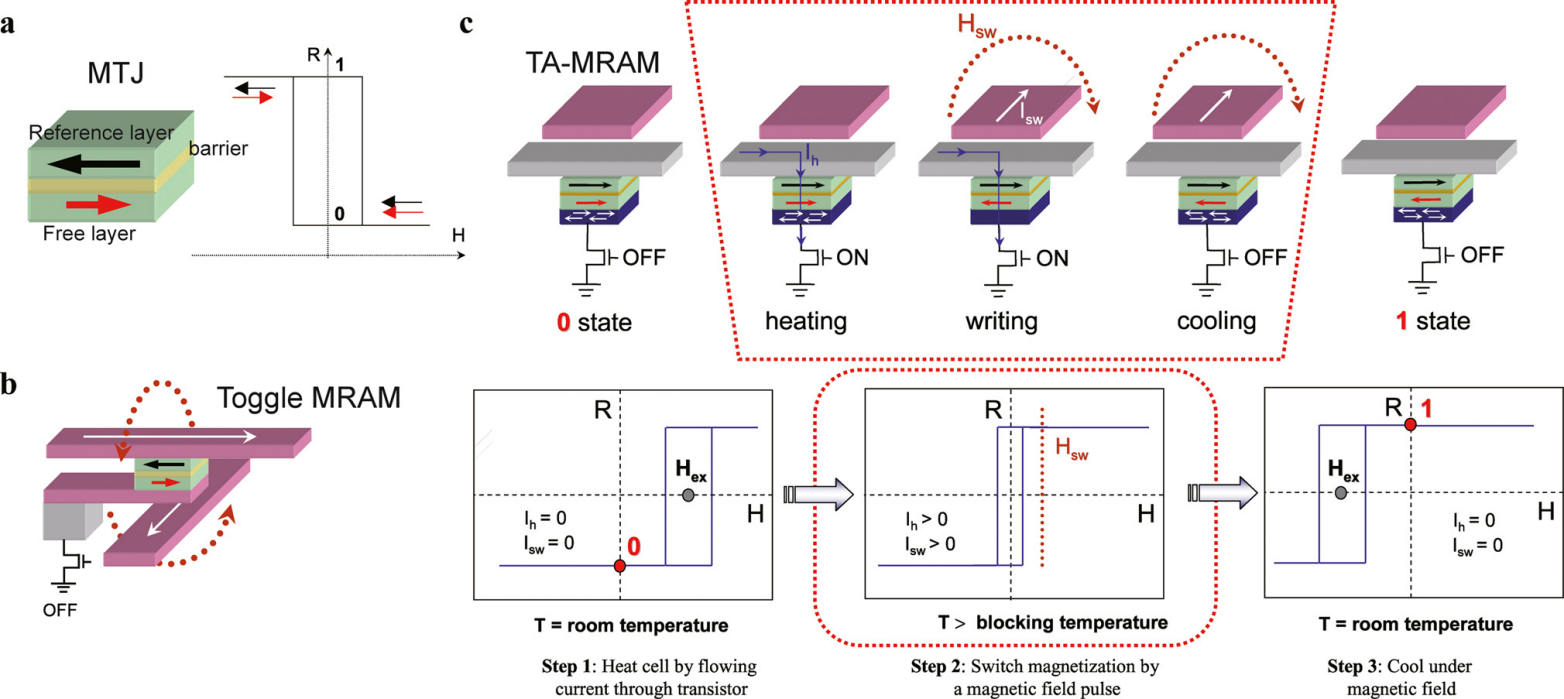
**MTJ, Toggle-MRAM, and TA-MRAM**. The schematic structures of the typical (a) MTJ and (b) Toggle-MRAM. The MTJ displays two resistance states for antiparallel and parallel. The red dotted lines present the Oersted magnetic field generated by the currents during the writing process of Toggle -MRAM. (c) The writing steps of the TA-MRAM [42].

Inspired by the structure of spin valves, AFMs have been widely used in MRAMs. In most current MRAM designs, the memory element is an MTJ [29], [30], [31], [32] that consists of two magnetic electrodes (the free layer: FL and the reference layer: RL) sandwiching an insulative tunnel barrier, as shown in Fig. 3a. The resistance of these MTJs depends on the relative orientation of the magnetic moments at the two magnetic electrodes interfacing with the tunnel barrier. When the magnetic moments of the two magnetic layers are antiparallel, the resistance of the tunnel junction is significantly higher than that when they are parallel.

The typical structure of Toggle-MRAM [33] is to arrange write lines above and below the MTJ unit, and the Oersted magnetic field generated by the current passing through these write lines realizes the write operation to the specific MTJ unit, as shown in Fig. 3b. The free layer is always a ferromagnetic layer with in-plane magnetic anisotropy which can be easily manipulated by an Oersted magnetic field. The reference layer is part of the trilayer known as the SAF [34]. The absence of net magnetization in the SAF prevents any impact on the free layer. An antiferromagnetic layer with strong interfacial exchange fields (such as IrMn [35] and PtMn [36]) was placed below the reference layer to pin the magnetic moment of the bottom SAF layer and ensure thermal stability.

SAF and AFM as the reference layer are also widely used in other generations of MRAMs, such as STT-MRAMs.

In 1996, Slonczewski [37] and Berger [38] theoretically proposed a purely electrical method for MTJ writing. The free layer can be switched using the spin current that can transfer the spin angular moment. Therefore, the torque generated by the polarized spin current is referred as STT. The basic structure of an STT-MRAM unit is an MTJ and the peripheral circuits. The reference layer is pinned in a certain direction, which can be achieved by the EB from the interface between the reference layer and the adjacent AFM layer. However, the reference layer pinned in a specific direction provides a net magnetic moment that can interact with adjacent MTJs and thus affects the performance of STT-MRAM. The interaction can be significant when the size of the MTJ shrinks to the nanometer scale. Similar to Toggle-MRAM, SAF was introduced to eliminate the net magnetization and closure the magnetic flux.

For in-plane magnetic anisotropy (IMA)-MRAM devices, the easy axis of the free layer is parallel to the moment of the pinned reference layer. The most practical origin of strong in-plane anisotropy is shape anisotropy. A rectangular or elliptical shape can ensure a sufficient anisotropic field. However, a large demagnetization field is simultaneously introduced, which increased the switching energy consumption. Moreover, shape anisotropy is unfavorable for advanced technology nodes; therefore, perpendicular magnetic anisotropy (PMA)-MRAM with circle-shaped MTJs is more suitable for high-density energy-efficient MRAM applications. However, the limitation of the circuit structure prevents MRAM with field-induced switching processes from using PMA-MTJs. Therefore, PMA-MTJs have attracted increasing attention since the invention of STT-MRAMs. The PMA in the MTJs relies on interfacial interaction [39,40]. In the HM/FM/MgO triple-layer, the interface of the ferromagnetic layer and adjacent heavy metal, such W, Mo, or Ta, can provide considerable PMA [41]. Bulk PMA can be achieved in multilayers, such as [Co/Pt] and [Fe/Pt]. However, the multilayer cannot match the requirement for the reference layer or free layer of high-TMR MTJs, which depends on the CoFeB/MgO/CoFeB triple-layer structure. Thus, the SAF is a promising candidate for the PMA STT-MRAM structure.

Both Toggle- and STT-MRAMs require one magnetic free layer with a magnetic moment that can be reversed easily, and another magnetic layer with a stable state. This demand was fulfilled by the AFM. EB can provide a strong interfacial interaction between the FM and AFM layer, while the SAF could also pin the reference layer via AFM-like coupling between two FM layers. AFM enables a practical MTJ structure, which is key to TMR reading and toggling or STT writing. The introduction of AFM removed the technological barrier of MRAM application.

### 2.3 AFMs in TA-MRAMs

In spin valves, Toggle- and STT-MRAMs, AFM was introduced to pin the FM reference layer. Moreover, AFM/FM interface coupling can be used to assist information storage in the free layer. Another new write approach for magnetic-field-switched MRAM is TA-MRAM [42], in which an AFM layer with a lower blocking temperature is coherent with the free layer and the AFM layer with a higher blocking temperature is coherent with the reference layer. The coupled FM and AFM layers are used for data storage, which can have good thermal stability and immunity to the external field owing to the application of the AFMs. The blocking temperature is a typical feature of AFM materials and is related to the disappearance of the EB field [42]. Usually, when the temperature is increased above the lower blocking temperature of the AFM, the magnetic anisotropy of the AFM disappears, which can be used as a writing program in TA-MRAM. As shown by the three steps for writing in TA-MRAM in Fig. 3c, when the temperature of the storage layer is increased by an applied current to a temperature higher than the blocking temperature of the top antiferromagnetic layer, the interaction between the AFM and FM layers weakens or disappears, and a very small Oersted magnetic field can switch the storage layer [42]. If the increased temperature of the devices is not higher than the blocking temperature, the strong coupling between AFM and FM in the free layer maintains the stability for the data storage.

Although TA-MRAM improves the thermal stability, write selectivity, and power consumption in MRAM, it also brings the inconvenience of simultaneous device integration [42]. The current paths for the heating and Oersted field are separated, which is complex for the writing program and limits the latency of the devices. Meanwhile, the power consumption of the TA-MRAM is not sufficiently optimized. Moreover, since the heat treatment requires a special process, considering the limitations of factors such as thermal conductivity, the choice of materials is restricted. All these shortcomings limit the use of TA-MRAM for further applications, leading to the next STT-MRAM. Based on the TA-MRAM designs, the concept of thermally assisted STT-MRAM has also been proposed [43]. The free layer was pinned using an adjacent AFM layer. The interaction between the FM and AFM was weakened by heating the free layer to the blocking temperature. The switching current could be decreased; thus, lower energy consumption and prolonged duration can be achieved. However, owing to the limitations of material selection, the joint effect of STT and thermally assisted switching has not become the mainstream technology for MRAMs. Nevertheless, SOT-MRAM has attracted extensive attention owing to its enhanced endurance, switching speed, and reliability. In SOT-MRAMs, AFMs, in addition to being used as pinning materials, show more promising application potential.

### 2.4 AFMs in SOT-MRAMs

Two main challenges limit the further development of STT-MRAM. The first challenge is the long incubation time [44,45]. The switching torque is small in the initial state and the switching time is sensitive to the initial polar direction of the ferromagnetic layer. Thus, the switching process is intrinsically stochastic, which is unfavorable for fast writing. The second challenge is low endurance [46,47]. The write current passes through the insulator barrier layer directly, which may lead to the breakdown of the insulator and reduce the endurance of the MTJs. This is a trade-off between the switching speed and endurance of the STT-MRAM. Moreover, other challenges, such as asymmetric writing [5] and miswriting of reading current also restrict its performance.

SOT-MRAM is promising for overcoming these challenges. The SOT switches the ferromagnetic layer with the spin generated by spin-orbit coupling (SOC). The spin Hall effect (SHE) [48] and Rashba-Edelstein effect (REE) [49] are considered the main origins of SOT. The SHE can convert the charge current into a pure spin current without a polarized ferromagnetic layer [50]. The generated spin current is then injected into the adjacent ferromagnetic layer, exerting an SOT that is strong enough to reverse the magnetization or drive domain wall motion. REE can generate spin density in an inversion-symmetry broken system [51]. The ferromagnetic free layer/SOT source layer interface automatically satisfies the requirement of non-centrosymmetry. The spin density is generated at the surface when an in-plane electric field is applied and then interacts with the magnetization in the free layer through exchange coupling. Both mechanisms mentioned above can produce spin with a pure electric method and avoid the current passing through the barrier layer.

### 2.5 Field-free SOT switching by AFMs

PMA is considered to achieve high storage density and has thus been widely studied. Several mechanisms have been proposed to provide an additional out-of-plane torque in the SOT switching process to tackle this issue [52], [53], [54], [55], [56], [57], [58]. Mostly evidenced is to apply an external in-plane magnetic field (along x-direction) [52], which, however, impedes the high-density integration of devices.

The EB at the AFM/FM interface can provide an effective magnetic field to achieve deterministic switching. In 2016, A. van den Brink et al. demonstrated that by capping a layer of IrMn over the PMA Co/Pt film [59], the exchange interaction could provide a sufficient effective magnetic field H_EB_ along the charge current direction. The existence of H_EB_ enabled the field-free reversal of the FM layer. Y. Lau et al. reported similar results in the same year [60]. They achieved field-free SOT switching in IrMn/CoFe(IMA)/Ru/CoFe(PMA)/Pt multilayers. The IMA CoFe was pinned by an EB, and the interfacial exchange interaction between the two CoFe layers provided the out-of-plane torque during the switching process.

**Fig. 4:**
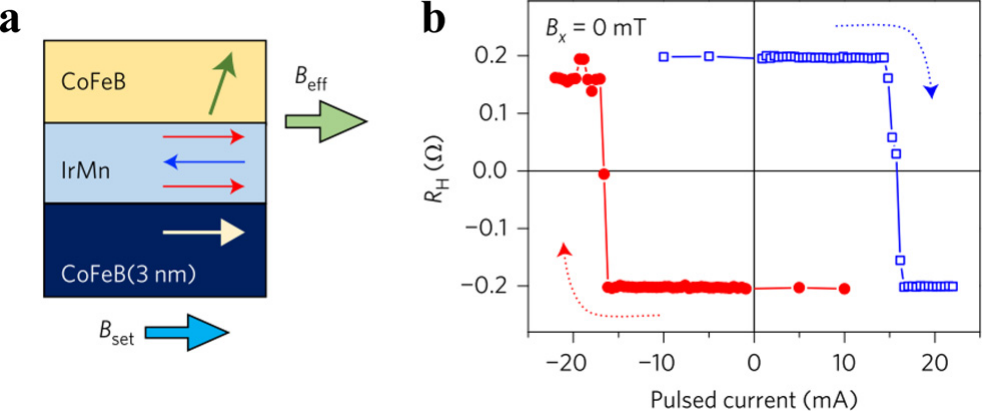
**AFM for field-free SOT switching.** (a). Schematic for the CoFeB(PMA)/IrMn/CoFeB(IMA) multilayers. The CoFeB at the bottom layer was introduced for enhancing the antiferromagnetic order of IrMn. The IrMn layer emerged an effective magnetic field to the CoFeB at the top layer. (b). Field-free SOT switching of the CoFeB(PMA) layer [55].

However, the AFM/FM/HM structure is not practical for SOT-MRAM manufacture. In 2016, S. Fukami et al. observed the FM layer switching without any applied external field in a [Co/Ni](PMA)/PtMn bilayer system on a Hall bar [54]. The SHE in AFM PtMn generates considerable spin current, while the in-plane EB at the interface of FM/AFM can simultaneously provide an effective in-plane field. Y. Oh et al. also achieved field-free FM layer switching in a CoFeB(PMA)/IrMn system on a Hall bar [55]. The switching in the CoFeB/IrMn bilayer was incomplete because of the weak in-plane EB. By introducing another layer of IMA CoFeB below the IrMn, the EB was enhanced by the IrMn/CoFeB(IMA) interface. The antiferromagnetic order can be more stable; therefore, there is a higher EB at the IrMn/CoFeB(PMA) interface. As illustrated in Fig. 4, complete field-free SOT switching was achieved in CoFeB(PMA)/IrMn/CoFeB(IMA) system. The AFM acted as an SOT source and the origin of the out-of-plane torque. Moreover, field-free SOT switching could also be achieved in the dual-SAF structure. J. Wei et al. demonstrated a field-free SOT switching in the PMA CoFeB/W/CoFeB SAF [61], where the W interlayer generated the SOT, and an adjacent IMA SAF layer provided the in-plane interaction, and therefore the out-of-plane torque. The process of AFM-induced field-free SOT switching is promising for reliable spintronic devices and facile device fabrication. In 2019, S. Peng et al. demonstrated field-free writing with ultralow power consumption by combining the SOT, EB, and voltage-controlled magnetic anisotropy (VCMA) effect [62]. The lowest writing energy was reduced to 6.2 fJ/bit, which illustrates the joint effect between SOT and EB, which is promising in the application of high-density and ultralow-power MRAM.

Recent studies have shown that out-of-plane SOT can be generated in materials with low symmetry, which has potential applications in PMA devices. For example, out-of-plane SOT was identified in WTe_2_/ferromagnet bilayers when a charge current was applied along the low-symmetry axis [63]. A similar phenomenon was observed in CuPt/CoPt multilayers [57].

Such an effect relies on crystal symmetry. Out-of-plane torque exists only when the symmetry is partially broken. A theoretical study [64,65] suggested that torque can be generated directly from the intrinsic SHE. Reducing the mirror or glide plane parallel to the crystalline axis can help achieve out-of-plane torque. Conventional heavy metals have a regular crystal structure, which is unfavorable for reducing the mirror symmetry. Such anomalous SOT is also absent in doped alloys, owing to the distribution of randomly disordered atoms.

AFMs can also give rise to out-of-plane torque because their highly symmetrical crystal structure is broken by magnetic moment. The non-collinear magnetic structure naturally results in relatively low symmetry. J. Zhou et al. observed out-of-plane torque in non-collinear AFM IrMn_3_ in 2020 [66]. L1_2_-IrMn_3_ has a uniform face-centered cubic structure with a Pm-3m space group. The triangular magnetization kept the 3-fold rotational symmetry of the [111] axis. However, only one magnetic mirror symmetry was retained along the z-axis for the (001)-oriented crystal. Therefore, when a charge current is applied to the low-symmetry axis, an out-of-plane torque can be detected by ST-FMR. Similar to IrMn_3_, the crystal structure of Mn_3_GaN without magnetization is also highly symmetric. The triangular magnetic moment broke most of the mirror planes parallel to the z-axis for the (001)-oriented crystal. In contrast to conventional SHE, the spin current generated in non-collinear AFM Mn_3_GaN has an out-of-plane portion, which finally results in an out-of-plane torque and could be favorable for field-free SOT writing [67]. Collinear AFMs can also generate anomalous SOT. Using L1_0_-IrMn as an example, J. Zhou et al. found that when the staggered magnetization is tilted away from the crystallographic axis, the magnetization breaks the crystal symmetry, and leads to an out-of-plane SOT [66]. Y. Jiang et al. theoretically studied the performance of AFM in SOT-MRAM by combining ab-initio calculation, micromagnetic, and macro spin simulation. The anomalous SHE in the IrMn system can reduce the threshold current density for writing, accelerate the writing speed and promote switching stability [68]. The results suggest that the anomalous SHE could expand the application of SOT, while AFMs showed great potential for enhancing the performance of SOT-MRAMs.

The origin of the out-of-plane SOT requires further discussion. Apart from the anomalous spin current generated from the intrinsic SHE, some other mechanisms were proposed to explain the experimental phenomenon.

X. Wang claimed that the anomalous SHE in the magnetic system originated from the interaction between spin Hall angle and the magnetic moment [69]:

(1) $$\theta_{ijk}^{SH}=\theta_{0}\in_{ijk}+\theta_{1}M_{l}\in_{iln}\in_{jlk}+\theta_{2}M_{l}\in_{ink}\in_{jln}$$

where $\in_{ijk}$ represents the Levi-Civita symbol, $M_{l}$ represents the magnetic moment, $\theta_{0}$ indicates the conventional spin Hall angle (SHA), $\theta_{1}$ and $\theta_{2}$ are anomalous SHE coefficient. Therefore, the polarization of the spin current generated by the SHE can be controlled by the magnetization or Néel order of the magnetization system.

M. Kimata et al. proposed the magnetic SHE (MSHE) to explain the anomalous sign change in non-collinear AFM Mn_3_Sn in 2019 [70]. The MSHE originates from the inter-band spin-density/electric-field response, and only exists in systems whose Bloch Hamiltonian is not time-reversal-invariant. The discovery of the MESH provides a new perspective for SHE manipulation. In 2021, X. Chen et al. detected an out-of-plane spin current in collinear AFM Mn_2_Au. The study proposed that the conventional carrier spin current can be rotated by a spin-orbit field, leading to an out-of-plane spin portion; this mechanism was described as the antiferromagnetic spin Hall effect (AFM-SHE) [71]. The AFM-SHE implies that out-of-plane SOT may exist in other collinear AFM systems with broken space-reversal symmetry.

**Fig. 5:**
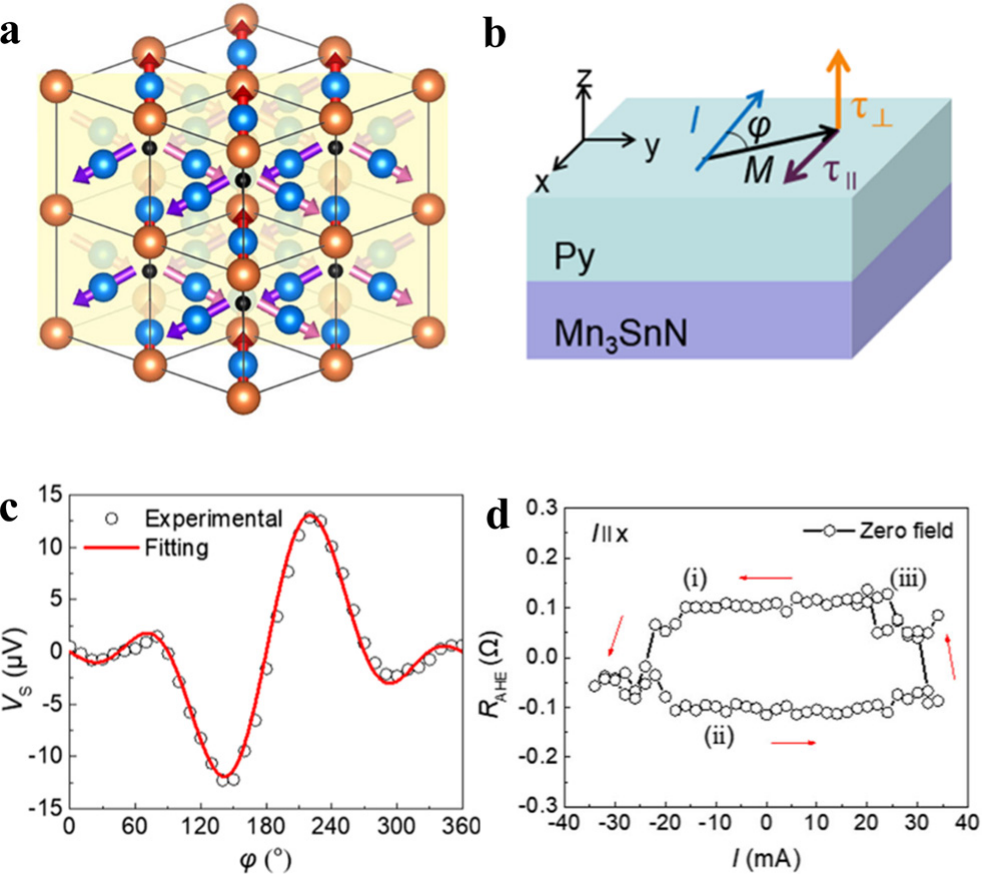
**Field-free SOT switching by the unconventional torque from Mn**_**3**_**SnN.** (a) Crystal structure of Mn_3_SnN, where the blue, orange, and black spheres represent the Mn, Sn, and N atoms, respectively. The yellow plane denotes the (110) plane. (b). Schematic diagram of Mn_3_SnN (110)/Py bilayers. (c). Angular dependence of line shape amplitude of ST-FMR signals for symmetric signals in the Mn_3_SnN/Py structure. The measurement result can be fitted by adding additional torque terms with the presence of out-of-plane spins. (d). Field-free SOT switching of Mn_3_SnN/[Co/Pd]_3_ system, where the switching is characterized by the AHE signal [72].

The out-of-plane spin current enriches the methods for field-free switching of PMA magnetization. Y. You et al. achieved field-free switching in a Mn_3_SnN/[Co/Pd]_3_ system [72]. As illustrated in Fig. 5, a considerable out-of-plane polarized spin current was observed by ST-FMR in (110)-oriented Mn_3_SnN and the magnetization switching in the Co/Pd multilayers was characterized by an AHE signal. A similar phenomenon was also observed in the Mn_3_Sn/[Ni/Co]_n_ system [73]. The switching current density of FM/Cu/Mn_3_Sn with the absence of an external magnetic field was much smaller than that of the magnetic-field-assisted FM/Cu/Ta system. These out-of-plane spins highlight the potential of AFM for high-density energy-efficient SOT-MRAM applications.

### 2.6 SOT efficiency of AFMs

To evaluate the strength of the SOT, SHA $\theta_{SH}$was used as the general experimental parameter. It is defined as the ratio between the spin current and the charge current. The SOTs of conventional heavy metals are usually damping-like torque generated from SHE, so $\theta_{SH}$ can well characterize the efficiency of charge-to-spin conversion. However, when the field-like torque is negligible, the experimentally measured result contains not only the contribution from the SHE but also the REE portion. In this case, the SHA is an effective parameter for describing the charge-to-spin conversion efficiency [74].

The SOT study of heavy metals showed that 5d heavy metals were able to achieve a high charge-to-spin conversion efficiency [48,75]. Therefore, metallic AFMs compounded by heavy metals may also be capable of comparable SHA. J. Mendes et al. first measured the SHA of metallic AFMs Ir_20_Mn_80_ using spin pumping in 2014 [76]. The $\theta_{SH}$ of polycrystal IrMn was estimated to be approximately $0.8\;\times \theta_{SH,Pt}$, which means that the charge-to-spin conversion efficiency of AFM is comparable to that of heavy metals. W. Zhang et al. measured the $\theta_{SH}$ of metallic AFMs $X_{50}Mn_{50}$ in 2014 [77], where X = Fe, Pd, Ir, and Pt. Spin pumping method was used. Their results showed that the $\theta_{SH}$ of these collinear AFMs varied from 0.008 to 0.06. The first-principles calculation results indicated that the scale of the spin current generated by AFMs changed significantly when the applied electric field or staggered magnetization was different. This implies that SHA can be promoted when the crystal structure is better. In 2016, Y. Ou et al. exhibited considerable SHA in FM/PtMn structures by spin-torque ferromagnetic resonance (ST-FMR) [78]. The SHA reached 0.16-0.19 when the FM layer is IMA. For the PMA FM/PtMn bilayer, the measured SHA was 0.11. When a thin Hf layer was inserted into the FM/AFM interface, a high SHA of 0.24 was observed in the work of FM/Hf/PtMn. W. Zhang et al. reported the facet-dependent SHA of AFM IrMn3 [79]. The magnetization of Mn ions was triangularly arranged in the (111) plane. The (001)-oriented crystal exhibits SHA of approximately 0.20, which is higher than that of the (111)-oriented films. A large SHA of 0.35 was achieved in (001)-oriented single-crystalline IrMn3 after annealing in a perpendicular magnetic field. The charge-to-spin efficiency was better than those of most heavy metals.

The first-principles calculation evidence and the conflict-measured results implied that the SHA of metallic AFMs could be strongly correlated with the phase or orientation of the AFM crystal. Polycrystalline AFM may lead to an average effect of SHA owing to the anisotropic SHE and randomly oriented crystal grains [77]. Furthermore, scattering at the interface of the domains may also reduce the efficiency of the SOT. In 2019, J. Zhou et al. deposited a well-defined (001)-oriented L1_0_-IrMn film on KTaO_3_ (001) substrates [80]. In contrast to bulk IrMn, the magnetization of the Mn ions was tilted away from the [001] direction by 56 degrees. An ultra-high SHA of 0.60 was measured by ST-FMR in Py/IrMn bilayers. However, the SHA reduced rapidly to 0.22 when a layer of Cu thin film was inserted. This was attributed to the broken interface exchange coupling, which indicates that the SOT may consist of a strong bulk SHE and comparable interface contribution. Moreover, extremely high SHA in noncollinear AFM IrMn_3_ of 1.01 was reported by Zhou et al. in 2020 [66]. The SHA of collinear L1_0_-IrMn and the gamma phase IrMn were measured as 0.61 and 0.80, respectively, which are also considerable and promising for practical applications.

### 2.7 Magnons in AFMs

It is noted that the spin current mentioned above is commonly associated with charge flow, signifying the unavoidable Joule heat and relatively short spin propagation length, typically on the order of nanometers [81]. Recently, another class of spin current, defined as the magnon current, has emerged and attracted significant attention [82]. The magnon current is associated with precessing spin moments rather than moving electrons, resulting in lower Joule heat dissipation. Moreover, the magnon current can flow for a long distance of up to several micrometers even in insulators [83], [84], [85], including easy-axis [86,87] and easy-plane AFM insulators [88,89]. Recently, Wang et al. studied the magnon torque in AFM NiO and observed magnon-torque-induced full magnetization switching in Bi_2_Se_3_/NiO/FM devices [90]. The measured magnon torque is comparable with the previously observed electrical spin-torque ratios. This study opens an avenue for magnon-based memory and logic with ultralow power consumption. For SOT-MRAM applications, increasing the magnon torque ratio is required to achieve low power dissipation, which may refer to pure magnon-driven magnetization switching without any electrical contribution.

The next-generation technology of SOT-MRAM requires a faster field-free switching speed, lower power dissipation, and higher integration density, in which AFMs may play an important role owing to the features of high intrinsic precession frequency, EB generation, high SOT efficiency, and absence of a stray field.

## 3 New mechanisms of the AFM manipulation and detection

Both the GMR heads and MRAMs rely mainly on the interfacial effect of the AFM to assist in data storage. However, information can also be recorded in AFM itself. Here, we introduce some physics mechanisms in AFM manipulation and detection, which can open an alternative avenue for AFM application in MRAM.

Just as AFMs can be utilized to generate spin currents for field-free switching in SOT-MRAM, they can also absorb spin currents. The interactions between the spins of conduction electrons and the local moments of the lattice, which are widely used in the electrical manipulation of ferromagnetic materials, can result in changes or dynamic excitations of the AFM spin structures via the STTs. Depending on the spin configurations, AFMs can be divided into collinear and non-collinear AFMs, and the mechanisms of electrical manipulation and detection vary in some respects.

### 3.1 AFM detection

#### 3.1.1 Collinear AFMs

The AMR effect was first found in 3*d* transition metals and alloys [91], and has been widely used as a detection element to read out memory states stored within a ferromagnetic metal, the resistivity of which depends on the orientation of the magnetization with respect to the direction of current flow or crystalline orientation [92] (the latter is known as the crystalline anisotropic magnetoresistance).

**Fig. 6:**
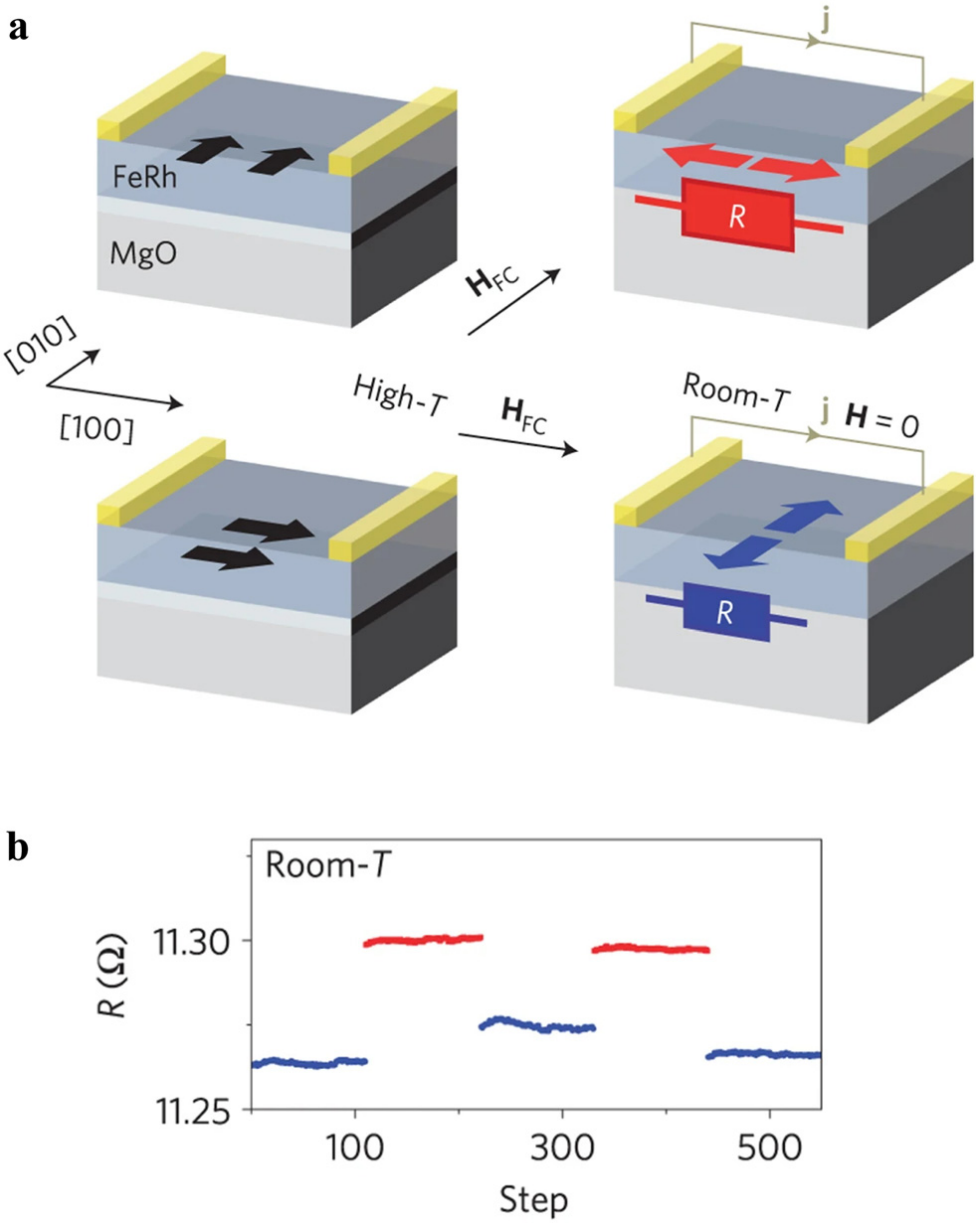
**AFM-AMR memory functionality in a FeRh resistor.** (a) Schematic illustration of the antiferromagnetic FeRh/MgO structure and of the memory writing and reading set-up. The antiferromagnetic Néel order is set toward two orthogonal directions [100] or [010] by cooling the sample in a field H_FC_ (either along the [010] or [100] crystal axis) from a temperature above the antiferromagnetic-ferromagnetic transition in FeRh to below the transition temperature. (b) AMR measured at room temperature after field-cooling the sample with H_FC_ parallel and perpendicular to the current direction [93].

Interestingly, the AMR is invariant upon magnetization reversal. Hence, AMR and related effects could also be used to detect the Néel vector states in AFMs, where AMR refers to the dependence of the resistance upon the relative orientation between the current flow and the AFM Néel vector. A straightforward method to characterize the AMR of both the FMs and AFMs is to apply and rotate a sufficiently large magnetic field. The resistivity is then measured as a function of the angle between the current and the magnetic field or the magnetization. This was demonstrated in collinear AFMs such as FeRh [93,94], CuMnAs [9], Mn_2_Au [95], and IrMn [96]. A typical example is the use of AMR in FeRh [93]. FeRh undergoes a metamagnetic phase transition from AFM to FM near room temperature. As shown in Fig. 6, by applying a magnetic field to saturate the FM order either along the [010] or [100] crystal axis at high temperatures and cooling to the AFM phase, the Néel order is set toward two orthogonal directions [100] or [010], perpendicular to the field-cooling direction. The AMR is then utilized to detect the Néel vector orientations; the Néel vector parallel (perpendicular) to the current flow corresponds to a high (low) resistance state.

Another important class of magnetoresistance phenomenologically similar to AMR is SMR. The SMR effect was first observed in magnetic multilayers containing a heavy metal adjacent to a ferromagnetic insulator (FMI) such as Pt/YIG [97]. The resistance of the HM depends on the angle between the magnetization and spin current polarization in the heavy metal, which is attributed to the combination of the SHE and inverse SHE in the heavy metal. The SMR enables remote electrical sensing of the magnetization direction in the FM, and since it remains unaffected by magnetization reversal, it could also be observed in bilayers consisting of an AFM adjacent to a heavy metal, where the SMR refers to the dependence of the resistance of the heavy metal upon the relative orientation of the Néel vector with respect to the direction of the SHE-induced spin current polarization. Indeed, it has been discovered for several AFMs such as insulating NiO in Pt/NiO/YIG trilayers [10,[98], [99], [100]] and Pt/NiO bilayers [101], insulating Cr_2_O_3_ in Cr_2_O_3_/W heterostructures [102], and metallic PtMn in PtMn/Pt stacks [103].

#### 3.1.2 Non-collinear AFMs

Non-collinear AFMs have attracted extensive attention owing to their nontrivial band structures. The electrical control and detection of non-collinear AFMs are significantly different from those of collinear AFMs.

**Fig. 7:**
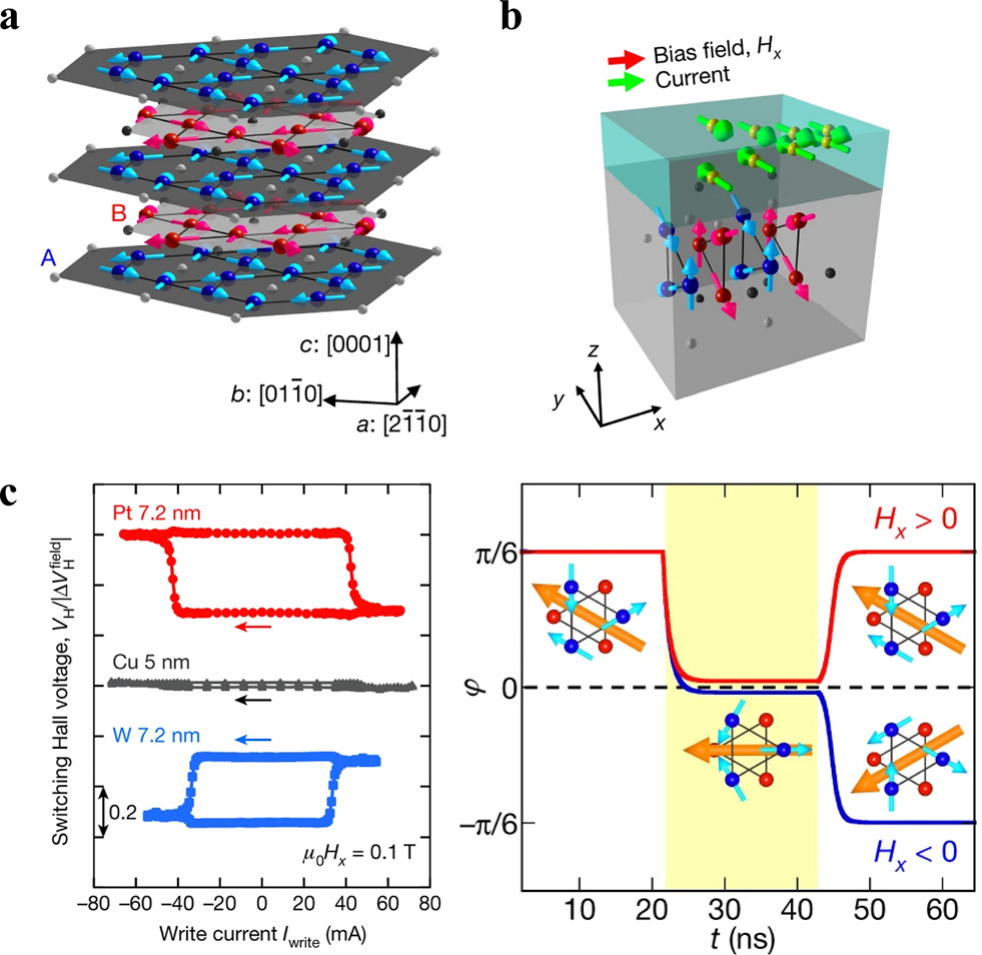
**SOT induced switching of a noncollinear AFM Mn**_**3**_**Sn.** (a) Mn_3_Sn crystal structure and inverse triangular spin structure. The large blue and red spheres (small grey and black spheres) represent Mn (Sn) atoms at z = 0 and 1/2, respectively. (b) Schematic of the spin-orbit torque switching of the magnetic octupole. (c) Measurements of spin-orbit torque switching for Mn_3_Sn/Pt, Mn_3_Sn/Cu and Mn_3_Sn/W and the model for the response of the magnetic octupole to the current and magnetic field [12].

For FMs with PMA, the AHE is most often utilized to detect magnetization switching [104]. Since AFM materials lack overall magnetization, common perception was that the AHE is absent in AFMs. This is true for most common collinear AFMs. However, for non-collinear AFMs whose crystal symmetry is broken, a nonvanishing Berry curvature is induced leading to the emergence of a finite AHE. This was discussed theoretically in stress-induced distorted γ-FeMn alloys [105] and AFMs with spins arranged on a Kagome-lattice such as IrMn_3_ [106], Mn_3_Ge, and Mn_3_Sn [107]. Subsequently, the corresponding experiments confirmed the large AHE in certain non-collinear AFMs. Mn_3_Sn [11] exhibits a large anomalous Hall conductivity of approximately 20 Ω^−1^cm^−1^ at room temperature and that of Mn3Ge [108] is approximately 50 Ω^−1^cm^−1^, which is the same order of magnitude as that of ferromagnetic metals. Fig. 7 shows the crystal structure of Mn_3_Sn, which has a stacked Kagome lattice of Mn atoms. An adjacent pair of the Kagome planes is called magnetic octupole. The orientation rotation of the magnetic octupole manipulated by the magnetic field applied within the Kagome plane could be monitored by the anomalous Hall voltage [12].

### 3.2 Manipulation by electrical current

#### 3.2.1 Collinear AFMs

SOT plays an important role in achieving the all-electrical isothermal manipulation of AFMs at room temperature. Until now, two main mechanisms, the bulk or interfacial inverse spin galvanic effect and the SHE, has been identified as the origin of SOT. In systems lacking (bulk or interfacial) inversion symmetry, the SOC becomes odd in momentum, which enables the electrical generation of a nonequilibrium spin density that gives rise to a torque and can be used for FM magnetization switching [49,109,110]. In contrast, the SHE utilizes the bulk spin-orbit interaction in a heavy metal to transfer a charge current into a spin current, which exerts a torque on the neighboring FM [111]. When an in-plane charge current is injected into a heavy metal, a spin current is generated and accumulates at the interface between the FMs and heavy metals. The directions of the charge current, pure spin current, and spin current polarization are mutually orthogonal.

**Fig. 8:**
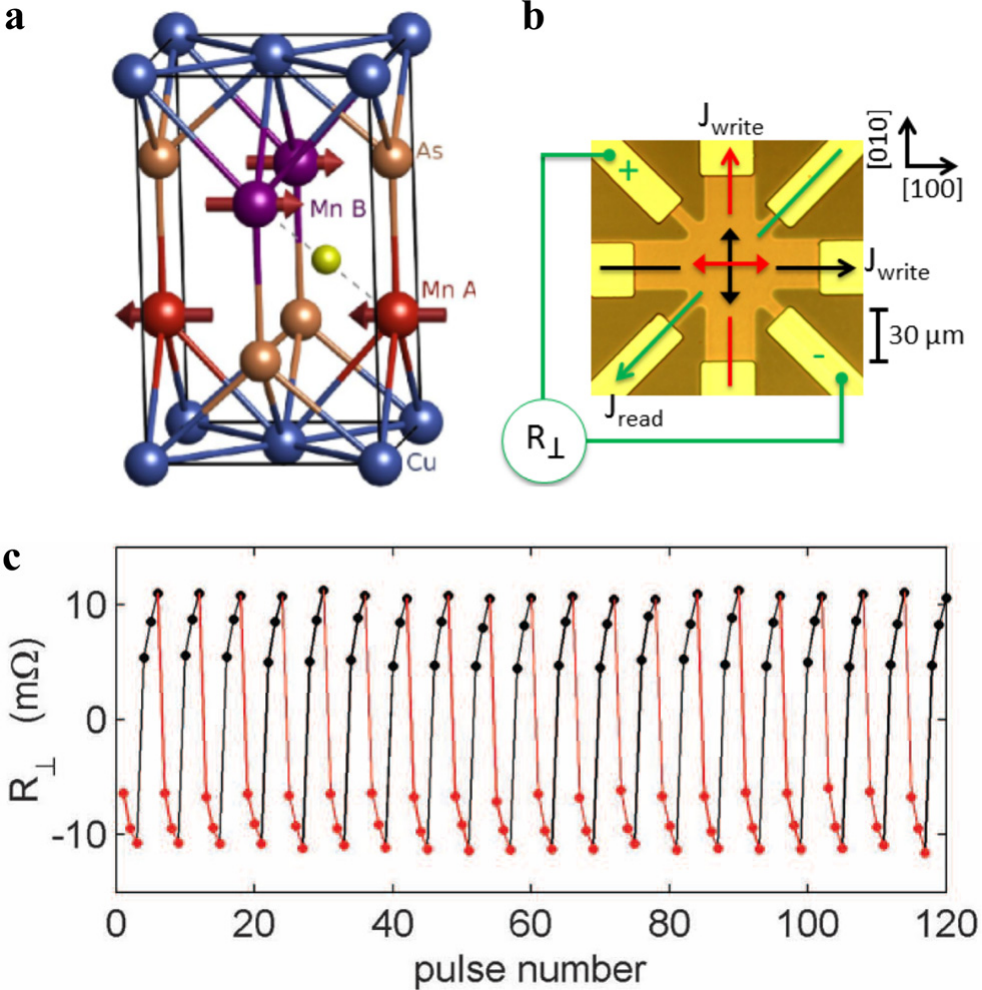
**Electrical switching of antiferromagnetic CuMnAs.** (a) CuMnAs crystal structure and antiferromagnetic ordering. (b) Optical microscopy image of the device and schematic of the measurement geometry. (c) Change in the transverse resistance after applying three successive writing pulses alternately along the [100] crystal direction of CuMnAs and along the [010] axis. The reading current *J*_read_ is applied along the [110] axis, and transverse resistance signals are recorded 10 s after each writing pulse. A constant offset is subtracted [9].

SOTs are also present in AFMs and could be utilized to manipulate the AFM Néel order. Early studies focused on AFMs with broken inversion symmetry manipulated by field-like Néel SOTs originating from the inverse spin galvanic effect. The basic idea of the relevant theoretical model proposed by J. Železný et al. is that for AFMs whose crystal structure is such that each crystal site for the two AFM spin sublattices has locally opposite inversion asymmetry, each sublattice experiences an opposite inverse spin galvanic effect [16]. This then stimulates opposite spin accumulation for each AFM spin, resulting in staggered field-like torques in both sublattices. AFM Mn_2_Au was first predicted to have the required crystal structure, of which each sublattice has broken inversion symmetry but together form “inversion partners” [16]. However, the first experimental observation of sublattice switching via Néel SOTs was demonstrated in CuMnAs, which possesses a similar crystal structure to that of Mn_2_Au with Mn sites forming locally non-centrosymmetric inversion partners as illustrated in Fig. 8 [9,112,113]. Wadley et al. demonstrated that the Néel order could be switched reversibly by 90° upon current injection, where the magnetic moments of Mn rotate to be perpendicular to the current, and this rotation could be detected via AMR. X-ray magnetic linear dichroism photoemission electron microscopy (XMLD-PEEM) measurements directly linked the AMR signals to the current-induced switching of AFM domains in CuMnAs. Moreover, Olejnik et al. demonstrated ultrafast reversible switching in CuMnAs with a speed scaled up to terahertz, two orders of magnitude faster than that of FMs switched by SOTs [114]. Subsequently, similar experimental results were reported for Mn_2_Au films with a high Néel temperature and better thermal stability than those of CuMnAs [115,116]. Current pulses induce reproducible switching of the AFM moments, which is detected by AMR and the planar Hall effect. Interestingly, thermal activation is highly significant in the electric-switching process of the Néel vector [117].

**Fig. 9:**
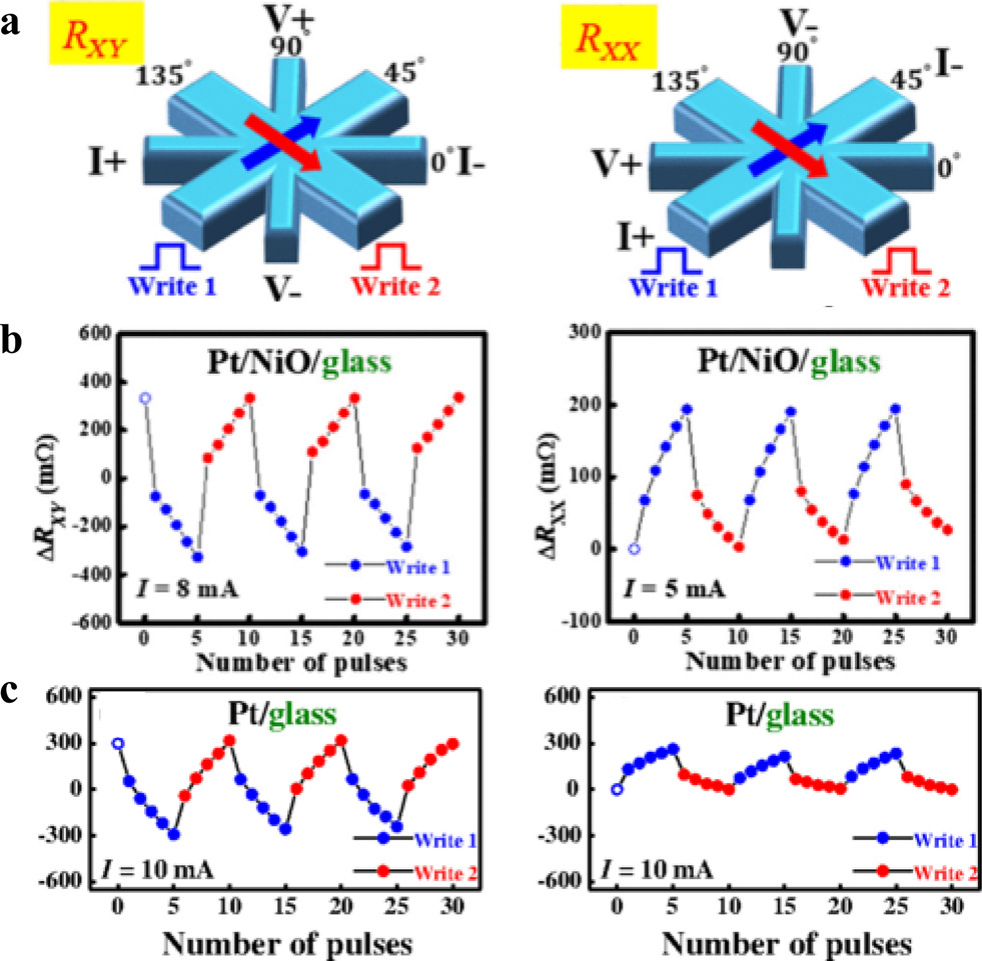
**Non-magnetic contribution in the electrical switching of an AFM.** (a) Schematics of the eight-terminal patterned structure with the pulsed writing current along two orthogonal directions for planar Hall and longitudinal resistance measurements. (b) Relative changes of Hall and longitudinal resistance in Pt/NiO/glass. (c) Similar switching behavior is observed without NiO [118].

Another promising method to electrically manipulate the AFM spin structure is to use the SHE arising from an adjacent heavy metal. For an FM structure with a net magnetization **M**, it is generally accepted that the SOT is composed of two components, namely, the damping-like torque $\tau_{\mathrm{DL}}\propto M\times \;\left(\sigma \times M\right)$ and field-like torque $\tau_{\mathrm{FL}}\propto M\times \sigma$, where σ is the polarization of the spin current. When the spin Hall torque is adopted to reorient the Néel order, the field-like torque exerted on each magnetic moment compensates for the exchange torque exerted by one sublattice on the other and thus cannot reorient the Néel order parameter. However, the damping-like torque, which is even in the magnetization direction, cants the magnetic moments but does not compensate for the exchange torque, and thus leads to a rotation of the Néel vector via the exchange torque [1]. Because reorienting the Néel order by damping-like SOT does not require that the spin sublattices form the inversion partners, Néel order switching was shown in a great variety of AFMs adjacent to a heavy metal. In AFM/heavy metal heterostructures, both the AMR and SMR depending on the relative angle between the Néel vector and the direction of the spin polarization induced by the SHE in the heavy metal layer can be used for the electrical detection of the Néel vector orientation. However, some reports have shown that the detected AMR/SMR signal could be a thermal or electromigration artifact arising from the high current required for the switching of the AFM state in a series of experiments in the structures with (Pt/NiO/glass) and without (Pt/glass) the AFM layer, as shown in Fig. 9 [118], which raises the challenge of unequivocally detecting the AFM Néel vector. Finally, by applying a magnetic field or changing the temperature, Y. Cheng et al. concluded that the sawtooth magnetoresistance signal is due to an artifact of Pt, while the actual spin-orbit torque-induced AFM switching is steplike in Pt/Fe_2_O_3_ systems [18], which further establishes the magnetic origin in resistance variation, as well as in Pt/CoO systems [19]. Meanwhile, some magnetic imaging techniques, such as XMLD-PEEM [20,119] and MOKE microscopy [120], have been employed to confirm the switching of AFM moments. Therefore, neglecting the weak signal, AMR/SMR could be an effective way to detect the direction of the AFM moments. As for the mechanism of the SOT induced AFM switching, several works have disclosed that both the current induced anti-damping (SOT) and the thermoelastic effect contribute to the switching of the AFM Néel vector. E. Cogulu et al. [20] carried out a direct observation image of the current pulse-induced AFM Néel vector switching by PEEM and claimed that both the spin-orbit torques and the purely thermal effects could help in the AFM reversal. Moreover, several harmonic tests [121], [122], [123] were carried out to further quantify the efficiency of the current-induced field-like torque and anti-damping torque, which could directly characterize and help in improving the SOT in AFM/heavy metal systems.

**Fig. 10:**
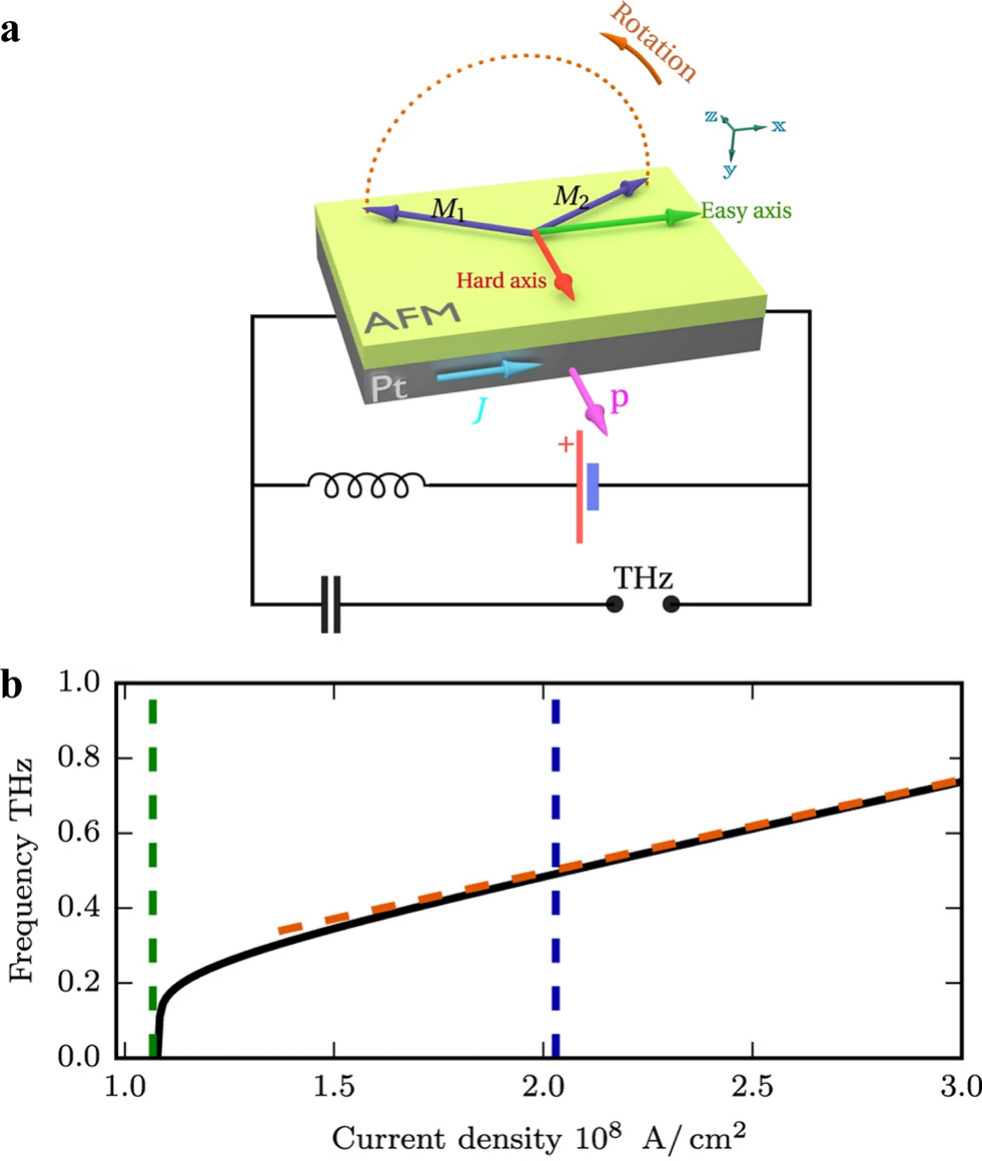
**THz-frequency oscillator based on a Pt/AFM bilayer.** (a) Schematic of THz-frequency magnetization dynamics based on a Pt/AFM bilayer. The hard axis lies in the bilayer plane parallel to the direction of spin polarization induced by the SHE in the Pt layer. The spin-torque induced non-uniform in time rotation of the canted antiferromagnetic sublattices creates a spin-pumping signal at THz frequency which is transformed into an electric field via the inverse spin-Hall effect in the Pt layer. (b) Calculated THz frequency as a function of the DC electric current density [124].

Current-induced switching and excitation of spin dynamics by spin-orbit torque are governed by the same physics. As discussed above, in AFM/heavy metal heterostructures, the damping-like torque exerted on the AFM cants the sublattice magnetizations and the internal exchange torque induces rotation of the sublattices which is non-uniform in time, as shown in Fig. 10 [124]. Consequently, a spin-pumping signal at the THz frequency is induced and subsequently transformed into an electric field via the inverse SHE in heavy metals. Another theoretical mechanism of the spin-torque triggering spontaneous excitation of THz oscillations was proposed by Cheng et al. [125]. In their study, the spin torque was renormalized by the backflow of the pumped spin current, which stabilized the AFM precession. For MRAM based on heavy metal/AFM structures, which will be discussed in the next section, spin-torque-induced AFM precession plays an important role.

#### 3.2.2 Non-collinear AFMs

Recently, H. Tsai et al. demonstrated the electrical switching of the anomalous Hall resistance of a polycrystalline non-collinear AFM Mn_3_Sn film using the same protocol for FMs [12]. The switching polarity is determined by the combination of the current direction, assistance magnetic field direction, and the spin Hall angle sign of the heavy metal which generates a spin current via the SHE. According to their provided model, the Kagome plane is perpendicular to both the film plane and applied current direction and the assistant bias field is along the current direction. The magnetic octupole was first rotated by π/6 to the state with an angle of φ=0±δ during the application of the write current. Upon turning off the current, the octupole polarization direction further rotates another π/6 or rotates back to the initial position, which is determined by the bias field polarity. Y. Takeuchi *et al.* revealed the unique response to spin-orbit torques in the same structure (Mn_3_Sn) [126]. A characteristic fluctuation of the Hall resistance under the application of an electric current was observed, which was attributed to SOT-induced continuous rotation of the chiral-spin structure. Electrical switching in non-collinear AFM Mn_3_Sn via spin-orbit torques offers a promising avenue for AFM spintronics.

### 3.3 Manipulation by electrical field

Apart from current-induced switching in AFM spintronics, the electric-field control of antiferromagnetism is also attracting attention owing to its advantages of low energy consumption. Piezoelectric materials have been widely employed to control the magnetic order. The lattice or shape of piezoelectric materials can be modulated by electric fields via the inverse piezoelectric effect, the strain generated by which can be transferred to proximate AFMs while they are integrated onto ferroelectric substrates, resulting in the modulation of antiferromagnetism [127].

H. Yan et al. demonstrated a piezoelectric strain-controlled AFM memory device that was insensitive to magnetic fields by depositing textured MnPt films on ferroelectric Pb(Mg_1/3_Nb_2/3_)_0.7_Ti_0.3_O_3_ (PMN-PT) substrates [128]. The main mechanism is that the AFM axis of the (101)-oriented grains rotates toward the sample surface normal under the biaxial compressive strain induced by the electric field. The two states are nonvolatile and can be switched by electric fields at zero field and are found to be robust under a strong magnetic field of 60 T owing to the strong AFM coupling in MnPt. Subsequently, X. Chen *et al.* utilized the ferroelastic strain from PMN-PT to switch the uniaxial magnetic anisotropy in AFM Mn_2_Au films with a small electric field at room temperature and showed that the asymmetric Néel SOT, and the corresponding AFM ratchet could also be reversed by electric fields [129].

Strain combined with electric fields induces additional magnetoelastic energy and can control the magnetic order of the AFMs. Some new mechanisms for the modulation of AFM have emerged and drawn much attention. Electric-field-controlled ionic liquid gating, compared with ferroelectric strain, has the same advantages and can penetrate deeper. In 2015, Y. Wang et al. reported the control of the exchange spring in IrMn AFMs using an ionic liquid as the gate electrode [130]. Reversible modulation of the EB was observed at different gating voltages. The origin of the phenomenon was suggested to be the change in magnetic anisotropy and carrier density in IrMn. SAF can also be modulated by ionic liquid gating. Yang et al. reported voltage modulation of the Ruderman-Kittel-Kasuya-Yosida interaction via ionic liquid gating in SAFs [131]. This modification was related to the disturbance of itinerant electrons inside the SAFs and the corresponding change in the Fermi level.

In addition to the methods mentioned above, electrostatic modulation using dielectric materials and electrochemical modulation using ionic migration could also be used to tailor antiferromagnetism. Specifically, S. Jiang et al. demonstrated the control of the magnetic properties of a layered AFM CrI_3_ by electrostatic doping and realized a transition from an AFM to a FM state under a zero magnetic field [132].

In summary, electric-field control of antiferromagnetism is a promising approach for achieving ultralow-power spintronic devices. There are more focused reviews, where the electric-field control of magnetism has been extensively described [127,133].

## 4 The birth of next-generation AFM-based device

### 4.1 SOT-induced EB switching

Another effective way to achieve AFM-based memory is to introduce an EB as an information carrier. The EB is the loop shift in the magnetic hysteresis of the FM material, which is caused by the interface exchange coupling and can be stable under high magnetic fields. Usually, the EB field resultes from the magnetic anisotropy of the AFM and is one of the typical features of AFMs that can represent the direction of the AFM moments, especially at the interface. Therefore, the electrical manipulation of the EB field is highly desirable for information storage.

The first observation of SOT-induced EB field switching was made by Lin's group in 2019 [134]. They performed SOT measurements of a Pt(2)/Co(0.1)/IrMn(8) (nm) trilayer and detected the direction of the FM magnetization in Co by the focused polar MOKE (FMOKE) technique. The magnetism of Co with a thickness of 1.2 nm was under PMA. The SOT generated by the heavy metal Pt not only switched the FM magnetization but also the EB field simultaneously.

**Fig. 11:**
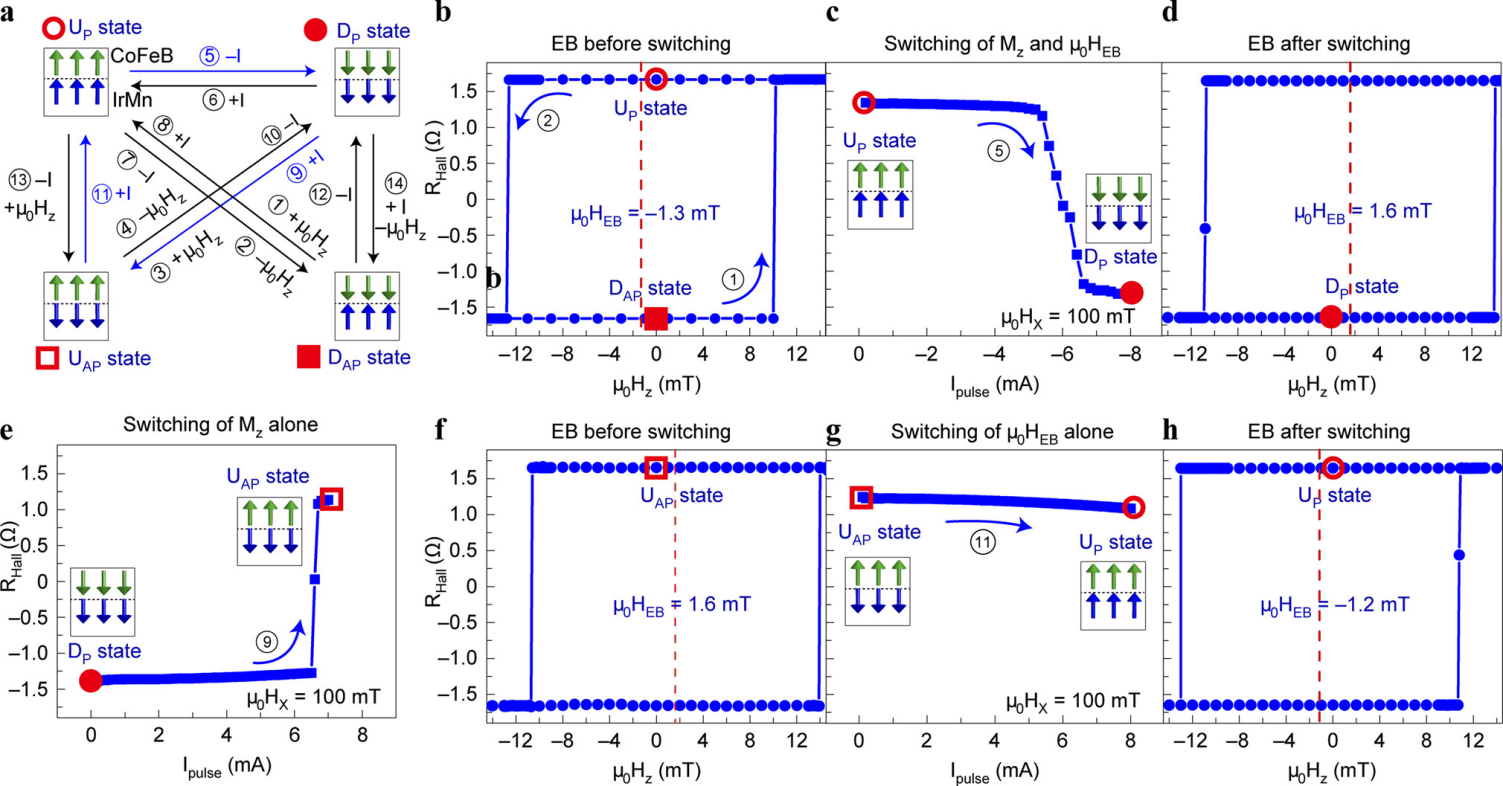
**SOT-induced EB switching in CoFeB/IrMn heterojunction.** (a) Schematic configurations. (b–d) EB and FM magnetization reversed at the same time. (e) The only switching of FM magnetization. (f)–(h) The only reversed of EB field. [22].

Peng et al. [22] conducted an in-depth study on SOT manipulation of the interface EB field in IrMn/CoFeB/MgO PMA heterojunctions. Apart from the literature in 2019, the SOT and EB were both generated by IrMn AFM. Further research revealed that the threshold current density of the switching EB field is higher than that of switching in FM magnetizations. By controlling the magnitude and direction of the SOT current, the magnetic moment of the FM layer and the EB field can be individually adjusted to obtain four different magnetic states, as shown in Fig. 11. Additionally, X-ray magnetic circular dichroism (XMCD) and polarized neutron reflection (PNR) measurements, as well as micro-magnetic simulations, revealed that the switching of the EB field in this system originates from the reversal of the uncompensated AFM pinning magnetic moment of the IrMn/CoFeB interface, which uncovers the physical mechanism of the electrical manipulation of EB in the AFM/FM heterojunction.

Meanwhile, the switching of EB in the IMA structure was also achieved by Kim et al. [135] in Pt/IrMn/CoFeB, which can be proposed in IMA-MTJs. Subsequently, many efforts have been made to electrically control EB fields in other structures such as IrMn/NiFe [136], Pt/Co/NiO [137], and AFM-Mn_3_AN/FM-Co_3_FeN bilayers [138]. Moreover, all-optical control of the magnetization and EB fields in IrMn/[Co/Pt]xN heterojunctions was realized by Vallobra et al. [139]. The helicity, fluence, and pulses of the laser determine the magnitude and sign of EB fields. Hence, the manipulation of the EB by various methods, such as electronics, lasers, and all-solid-state Li-ion redox capacitors [140], benefits the development of AFM-based applications in spintronics.

### 4.2 EB-based memories

Electrical manipulation of the EB field provides a new candidate for information storage, which may generate multilevel states and have potential applications in neuromorphic computing. Yun et al. [141] demonstrated that SOT can engineer double-biased hysteresis loops in an exchange-biased Ta/Pt/Co/IrMn/Ta system, which results in multilevel remanence through SOT-induced domain wall motion in the presence of an in-plane field. Meanwhile, another recent study [142] also reported that the SOT can reconfigure the direction of the EB field, thereby enriching the electrical and programmable operations of multilevel DW memory without the assistance of the fields.

**Fig. 12:**
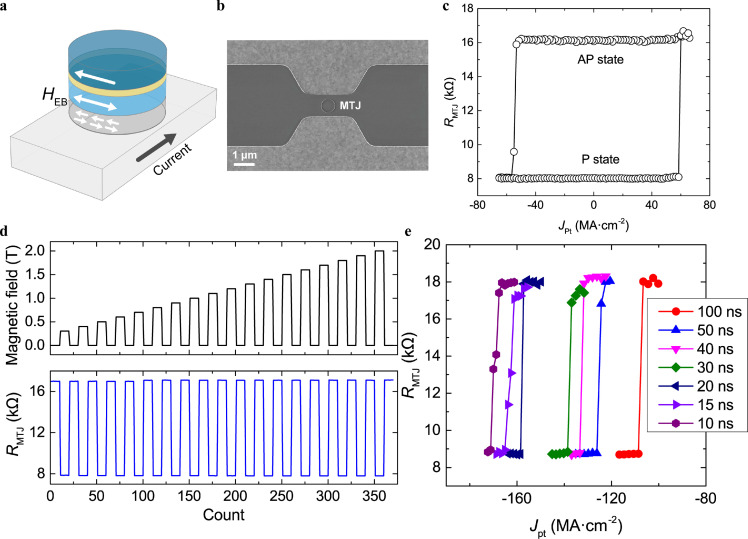
**Device structure and electrical performance of EB-MRAM.** (a) The schematic structure and (b) the scanning electron microscopy from the top-view of the devices. (c) The field-free SOT induced switching loop of the device. Two magnetic states are observed and the TMR ratio is over 100%. (d) The resistance response to the magnetic field up to 2 T. (e) The switching curves under different write pulse widths down to 10 ns. [21].

However, the read-out of EB-based DW memory is still focused on the AHE or Kerr signals, which also limits the application of AFM in memory. To overcome this problem and achieve an effective read-out of AFM is still a research interest in AFM and MRAM. Therefore, by combining the EB field with the TMR effect, a new data storage and writing method was discovered by Zhu et al. [21], which is expected to further improve the data storage density. Fig. 12a, b shows the proposed new type of three-terminal IMA-MRAM. The on-off ratio was greater than 100%, as shown in Fig. 12c. The EB field of the AFM/FM interface is adapted in the device instead of the shape anisotropy for data storage, and can stably maintain data even after the interference of an external magnetic field up to 2 T (Fig. 12d), thereby greatly improving the storage density of in-plane devices. Moreover, the achievement of the 10 ns field-free switching (Fig. 12e) and a good endurance over 10^10^ could address the challenges of the current SOT-MRAM. The macro-spin modeling revealed that the SOT-induced precession of the IrMn, as well as thermal assistance, could be responsible for the observed switching. The results highlight that the proposed EB-MRAM could have broad practical application prospects in high-density data storage and ultrafast computing which opens a new era from interesting but useless to interesting and very useful.

## 5 Conclusion

After nearly 25 years of development, AFMs have been widely used in both GMR read-heads and conventional MRAMs. Nonetheless, there are studies on AFM manipulations that try to optimize the use of AFMs as information carriers in electronic devices. However, the technical route of AFM-based memory remains unclear. Based on our investigation, we propose EB-MRAM as a potential next-generation AFM-based memory and provide some personal remarks.

The interlayer coupling between the AFM and FM was uncovered in spin valves, Toggle- and STT-MRAMs. The possible research point of SAF may be high-frequency precession signals, which may have potential applications in spin-torque nano-oscillators (STNOs) that have been studied by several groups recently [143]. Spin-polarized AFMs are complex and may introduce novel properties. Many efforts have been made to focus on the different crystal-symmetry dependences of the spin polarization in various metallic AFM materials, which may have obvious advantages in realizing field-free switching in SOT-MRAMs. On the other hand, the characteristics of the spin-polarized direction in AFM insulators such as NiO and Cr_2_O_3_, could also be interesting for specific structures in MRAMs [144]. Finally, it might take time for SOT in AFMs to be fully applied in practical memory devices.

SOT-induced switching of the EB in the AFM/FM interface was proven to be feasible. However, many features need to be deeply understood, such as the dynamics of SOT-induced AFM switching, the effect of heating during the switching, the writing speed of the EB, the size limitation, and the thermal stability of the devices. Furthermore, the reduction of data-writing power consumption in the EB-MRAM is of great significance, which could be optimized by the film stack design or by introducing the interplay among multiple effects, such as the STT or the VCMA. Finally, the realization of a full AFM tunnel junction based on a proper barrier is a long-term research target for AFM-MRAMs.

In summary, we described in detail the main applications of AFMs in GMR read-heads and the conventional MRAMs, reviewed the typical read-out and writing operations of AFMs, introduced an EB-MRAM to realize the detection of the magnetic moment in AFMs, and finally discussed the perspectives in AFM-based memories. Although the EB-MRAM is still under investigation, it is expected to promote the application of a new generation of AFM-based memories in the areas of massive data storage and ultra-high-speed information computing.

## Declaration of Competing Interest

The authors declare that they have no conflicts of interest in this work.
